# Vitamin D and subclinical cardiac damage in a cohort of kidney transplanted patients: a retrospective observational study

**DOI:** 10.1038/s41598-020-76261-5

**Published:** 2020-11-05

**Authors:** C. Alfieri, S. Vettoretti, O. Ruzhytska, M. T. Gandolfo, D. Cresseri, M. Campise, L. Caldiroli, E. Favi, V. Binda, P. Messa

**Affiliations:** 1 Nephrology, Dialysis and Renal Transplantation, Fondazione IRCCS Ca’ Granda – Ospedale Maggiore Policlinico – Milano, Via Commenda 15, 20122 Milan, Italy; 2 Department of Clinical Sciences and Community Health, University of Milan, Milan, Italy; 3 Department of Internal Medicine n3, Ternopil National Medical University, Ternopil, Ukraine; 4 Renal Transplantation, Fondazione IRCCS ca’ Granda Ospedale Maggiore Policlinico, Milan, Italy

**Keywords:** Nephrology, Kidney diseases, Chronic kidney disease

## Abstract

In 178-kidney transplanted patients (KTxp), the prevalence of hypovitaminosis-D, the presence and novel development of left ventricular hypertrophy(LVH) and the correlations between native Vitamin-D (25OHD) and LVH were evaluated during the 1st year of transplantation (KTx). Clinical and instrumental data were recorded at pre-KTx and at one (T1) and 12 (T12) months after KTx. 25OHD levels were considered sufficient (s25OHD, ≥ 30 ng/dL) or insufficient (i25OHD, < 30 ng/dL). 25OHD correlated at T1 with parathormone(PTH), and at T12 with 25OHD-T1 and PTH-(T1,T12). At T12, s25OHD (15%) had higher 25OH and alkaline phosphatase (ALP), lower Ca, at T1, and lower PTH-(T1, T12) than i25OH-T12. At T1, KTxp with LVH (LVH-T1pos, 42%) were older and with longer dialysis vintage than LVH-T1neg. At T12, KTxp with LVH (LVH-T12pos, 53%) were older, with higher systolic blood pressure (SBP) at T12 than LVH-T12neg. No relation between 25OHD and LVH were found. Novel LVH was found in 14% of KTxp. They were older, had higher SBP-T12 and lower serum albumin-T12 than the others. LVH-modifications and 25OHD were not correlated. Hypovitaminosis-D is highly prevalent in KTxp. LVH correlates with different risk factors according to the time elapsed from KTx. However, during the 1st year of KTx, no relationship between LVH and 25OHD was observed.

## Introduction

Patients affected by chronic kidney disease (CKD) have higher cardiovascular morbidity and mortality than the general population^1,2^. Data present in the literature report a significant and progressive increase of cardiovascular risk (CVR) in the presence of a reduced renal function. In the CKD population, CVR reaches its maximal impact in end stage renal disease (ESRD) patients.

Many traditional CVR factors are nowadays well characterized and when possible treated in clinical practice^3^. Among them, left ventricular hypertrophy (LVH), a marker of subclinical cardiac damage, is highly prevalent in CKD patients and has a strong impact in increasing their CVR^4^.

Recently, some “not traditional CVR factors” have been identified and are the object of studies and debates. Among them the role of vitamin D in modulating CVR has been the object of intense interest. Low native vitamin D (N-VitD) levels (25OHD < 30 ng/dL) are frequently found in CKD patients both before and after kidney transplantation (KTx)^5^. In addition to its well-known effect on mineral metabolism (MM), an implication for N-VitD has been hypothesized in the modulation of cardiovascular, metabolic, immunologic, neoplastic and infectious processes^6^. N-VitD deficiency has been related to LVH presence both in the general and in CKD populations, even if interventional studies are still missing^7^.

Although KTx is considered the best therapy for CKD patients, they are still burdened by a high CVR, potentially related to their clinical history of CKD as well as to some KTx specific risk factors^8^. In this context, the role of N-VitD status in the period following KTx has not been well characterized.

In this retrospective observational study, we evaluated a cohort of KTx patients (KTxp) aiming to explore:

1) the prevalence of N-VitD insufficiency at 1 month (T1) and 12 months (T12) after KTx;

2) the prevalence and the factors related to LVH;

3) the possible relationship between N-VitD status and LVH during the first 12 months after KTx.

The study hypothesis were: 1) The prevalence of low levels of native vitamin D in KTxp, from the early KTx period is high; 2) Several factors, including N-VitD status might be related to LVH; 3) Vitamin D status and/or levels have a relationship with LVH modifications during the first year of KTx.

## Results

### Overall cohort studied characteristics

The general characteristics of the studied cohort are summarized in Table 1. The median age was 49[39–59] years and the overall cohort was composed mainly of males (n = 118).

**Table 1:** cohort characteristics at baseline.

Parameter	
Number of patients (n)	178
Gender (n) (M/F)	118/60
Basal nephropathy n (%)	
GNC	38 (19)
ADPKD	38 (19)
Immun GNF	25 (12)
Vasculitis	8 (4)
CPNF/interstitial	13 (6)
HUS	2 (1)
Other	36 (18)
Not available	18 (9)
Type of dialysis n (%) (No/HD/PD)	20/116/42 (11/66/23)
Dialysis vintage (mths)	42 [16–73]
Age at KTx (years)	49 [39–59]
Pre-KTx diabetes n (%)	9 (5)
Kind of transplant n (%) (deceased donor/living donor)	144/34 (81/19)

Continuous variables were expressed as median value and interquartile range [25%ile; 75%ile].

*HD* hemodialysis; *PD* periotoneal diaysis; *GNC* chronic glomerulonephritis; *ADPKD* autosomal dominant polycystic kidney disease; *Immun GNF* Immunological glomerulonephritis; *CPNF* chronic pyelonephritis; *HUS* hemolytic uremic syndrome.

Most of KTxp (66%) had been treated with hemodialysis before KTx, whereas 20 KTxp (11%) received KTx before dialysis initiation. Dialysis vintage was 42 [39–59] months. Concerning basal nephropathy, in most of patients a chronic glomerulonephritis or an autosomal dominant polycystic kidney disease (ADPKD) was present. Immunological glomerulonephritis was found in 12% of KTxps. One hundred-forty-four patients (81%) received a graft from a deceased donor.

Both at one month (T1) and at 12 months (T12) after KTx, immunosuppressive therapy was mostly composed of steroids (cumulative steroids during the first year: 2732[2627–3074] mg, calcineurin inhibitors (cyclosporine or tacrolimus) and mycophenolate/mycophenolic acid.

N-VitD supplementation prescription was found only in 22 (12%) and in 37 (20%) KTxp at T1 and T12 respectively. Concerning antihypertensive therapy, both at T1 and T12, the majority of patients required at least two antihypertensive drugs to obtain adequate blood pressure control. The distribution of the immunosuppressive therapy as with the number of antihypertensive drugs was not significantly different between T1 and T12.

In Table 2 we reported anthropometric and biochemical characteristics of the overall cohort at the pre-KTx time and at T1 and T12.

**Table 2:** Anthropometric and biochemical characteristics of the overall cohort at the Pre-KTx time, at T1 and T12.

Parameters	Pre-KTx	T1	T12	p*
Body weight (Kg)	NA	66[57–75]	70[60–78]	** < 0.0001**
BMI (kg/m^2^)	NA	23[21–25]	24[22–27]	** < 0.0001**
SBP (mmHg)	NA	130[120–145]	130[120–140]	0.46
DBP ((mmHg)	NA	80[75–90]	80[70–85]	0.11
s-Creatinine (mg/dL)	8.1[6.1–10.9]	1.40[1.10–1.70]	1.36[1.15–1.60]	**0.04**
Blood glucose (mg/dL)	90[80–104]	83[82–93]	84[77–96]	0.93
Hb (g/dl)	11.1[10.6–12.2]	11.1[10.1–12.0]	12.7[11.8–13.6]	** < 0.0001**
s-Albumin (g/dL)	4.0[3.7–4.2]	4.3[4.0–4.5]	4.5[4.3–4.7]	** < 0.0001**
PTH (ng/mL)	228[132–390]	60[39–100]	56[37–82]	0.26
Ca (mg/dl)	9.15[8.7–9.6]	9.70[9.30–10.1]	9.80[9.50–10.1]	**0.02**
P (mg/dL)	5.1[4.4-]5.6	2.40[1.90–2.90]	3.10[2.70–3.50]	** < 0.0001**
ALP (U/dL)	82[62–141]	84[68–116]	79[63–107]	**0.05**
25OHD (ng/mL)	NA	13[8–19]	16[10–24]	** < 0.0001**
m25OHD (ng/mL)	NA	NA	15[11–20]	NA
Prot-U (g/24 h)	NA	0.20[0.15–0.30]	0.17[0.12–0.26]	0.60

Bold format: statistical significance (p < 0.05); t-test and Mann–Whitney test were used.*T1 vs T12.

Continuous variables were expressed as median value and interquartile range [25%ile; 75%ile].

*BMI* body mass index; *SBP* systolic blood pressure; *DBP* diastolic blood pressure; *Hb* hemoglobin; *PTH* parathormone; *Ca* calcium; *P* phosphorus; *ALP* Alkaline Phosphatase Prot-U: daily urinary protein excretion; “*s*” serum; *m25OHD* mean levels of native vitamin D; *NA* not applicable.

During the first year of KTx there was a significant increase of body weight and body mass index (BMI). However, most of the subjects had a BMI compatible with normal weight. Average blood pressure values were well controlled at both T1 and T12. During the first year of KTx we observed a significant reduction of serum creatinine (s-creatinine) whereas there were no significant differences in daily urinary protein excretion (Prot-U). Hemoglobin, Calcium (Ca), phosphorus (P), serum albumin (s-albumin) increased progressively between T1 and T2. Although a significant increase of 25OHD levels was found during the first year of KTx, average N-VitD concentrations were under the sufficiency cutoff level at both T1 and T12. Average parathormone (PTH) values were similar at both time points.

### Vitamin D status

#### N-VitD at T1

At T1, average 25OHD was 13 [18–19] ng/dL. Patients with sufficient 25OHD levels (s25OHD-T1) were only 2 KTxp whereas in 22 (12%) was prescribed N-VitD supplementation. At univariate regression, 25OHD-T1 levels correlated only with PTH-T1 (p < 0.0001). We did not find any influence on N-VitD status for gender, basal nephropathy, type of dialysis, presence of diabetes before KTx and immunosuppressive therapy.

#### N-VitD at T12

At T12, average 25OHD levels were still low in the overall cohort 16 [10–24] (p < 0.0001 vs 25OH-T1). At univariate regression, 25OHD-T12 was directly correlated with 25OHD-T1 (p < 0.0001) and inversely correlated with PTH-T1 and PTH-T12 (p < 0.0001 and p = 0.01 respectively). In 27 (15%) KTxp there was a significant increase of s25OHD status. In Table 3 we report the comparison between patients with insufficient 25OHD levels (i25OHD) and s25OHD KTxp at T12. We found that s25OHD had lower PTH both at T1 and T12 and Ca-T1 (p = 0.01, p = 0.002 and p = 0.02 resp.). We did not find any difference between i25OHD-T12 and s25OHD-T12 regarding renal function and in Prot-U. Categorical analyses did not demonstrate any influence of gender, dialysis characteristics, type of KTx and immunosuppressive therapy, cumulative steroid included, on vitamin D status at T12.

**Table 3:** General characteristics of the cohort according to N-VitD status at T12.

Parameters	i25OHD-T12 (n = 151)	s25OHD-T12 (n = 27)	p
Age (years)	49[39–59]	50[40–58]	0.82
Dialysis vintage (months)	44[17–64]	23[10–59]	0.17
Body weight—T12 (Kg)	69[59–84]	70[61–82]	0.57
BMI—T12 (kg/m^2^)	23[21–25]	24[22–27]	0.89
SBP-T1 (mmHg)	130[120–145]	140[130–145]	0.33
DBP-T1 (mmHg)	80[75–90]	80[80–90]	0.19
SBP-T12 (mmHg)	130[120–140]	125[120–140]	0.14
DBP-T12 (mmHg)	80[70–85]	80[70–85]	0.62
s-creatinine-T1 (mg/dL)	1.40[1.10–1.70]	1.30[1.10–1.65]	0.53
s-creatinine-T12 (mg/dL)	1.40[1.15–1.60]	1.35[1.10–1.45]	0.38
Blood glucose-T1 (mg/dL)	84[72–93]	79[76–90]	0.37
Blood glucose-T12 (mg/dL)	84[77–96]	85[77–94]	0.95
Hb-T1 (g/dL)	11.1[10.0–12.1]	11.1[10.4–12.0]	0.86
Hb-T12 (g/dL)	12.7[11.7–13.5]	13.2[12.0–14.0]	0.28
s-albumin-T1 (g/dL)	4.3[4.1–4.5]	4.05[3.9–4.4]	**0.02**
s-albuminT12 (g/dL)	4.5[4.3–4.7]	4.5[4.2–4.7]	0.98
PTH-T1 (ng/mL)	67[41–104]	41[34–65]	**0.01**
PTH-T12 (ng/mL)	60[40–84]	37[27–60]	**0.002**
Ca-T1 (mg/dl)	9.7[9.3–10.1]	9.6[8.8–10.0]	**0.02**
Ca-T12 (mg/dl)	9.8[9.5–10.1]	9.6[9.4–9.9]	0.09
P-T1 (mg/dL)	2.3[1.9–2.9]	2.5[2.0–2.9]	0.74
P-T12 (mg/dL)	3.1[2.7–3.5]	3.1[2.7–3.6]	0.56
ALP-T1 (U/dL)	87[69–116]	73[61–137]	**0.02**
ALP-T12 (U/dL)	80[61–108]	79[63–106]	0.37
25OHD-T1 (ng/dL)	12[8–17]	19[11–24]	**0.003**
25OHD-T12 (ng/dL)	15[10–20]	37[32–44]	** < 0.0001**
m25OHD (ng/dL)	14[10–18]	27[23–33]	** < 0.0001**
Prot-U T1 (g/24 h)	0.21[0.15–0.30]	0.24[0.16–0.31]	0.85
Prot-U T12 (g/24 h)	0.17[0.12–0.25]	0.19[0.13–0.36]	0.73

Bold format: statistical significance (p < 0.05); t-test, Mann–Whitney test were used.

Continuous variables were expressed as median value and interquartile range [25%ile; 75%ile].

*BMI* body mass index; *SBP* systolic blood pressure; *DBP* diastolic blood pressure; *Hb* hemoglobin; *PTH* parathormone; *Ca* calcium; *P* phosphorus; *ALP* Alkaline Phosphatase Prot-U: daily urinary protein excretion; “*s*” serum; *m25OHD* mean levels of native vitamin D.

### Left ventricular hypertrophy

All the 178 KTxp were studied by echocardiography (EC) to evaluate the presence of LVH at T1. EC was repeated for usual clinical surveillance and without a specific clinical indication in 75 KTxp at T12.

Both at baseline and after 12 months the prevalence of LVH and the distribution of the different cardiac geometric patterns were similar. In particular, the concentric pattern of remodeling (C-LVH) was the most prevalent (26% of the cohort studied) both at T1 and T12.

#### LVH at T1

At T1, average left ventricular mass index (LVMI) was 47.5[40–60] g/m^2,7^. At univariate regression, LVMI correlated directly with age (p = 0.03), dialysis vintage (p = 0.009), systolic blood pressure (SBP) (p = 0.01), BMI (p = 0.007), Prot-U (p = 0.02) and, inversely, with pre-KTx albumin levels (p = 0.01). LVH was found in 75 (42%) KTxp. The most prevalent LVH geometric pattern was C-LVH, that was found in 46 (26%) KTxp.

A comparison of the characteristics of the KTxp in whom echocardiography was performed both at T1 and at T12 according to the presence (LVH-T1pos) or the absence (LVH-T1neg) of LVH was performed.

LVH-T1pos were older than LVH-T1neg (p = 0.04). We did not find any difference between the two populations regarding BMI and renal function although Prot-U was slightly higher in LVH-T1pos (p = 0.04). We did not observe any significant difference in 25OHD or in other parameters of mineral metabolism. Categorical analyses did not show significant differences in: N-VitD status, gender, type of dialysis, type of KTx and immunosuppressive therapies between LVH-T1pos and LVH-T1neg.

In multivariate regression model (LVMI-T1 as dependent variable; dialysis vintage, BMI-T1, preKTx-albumin and pre-KTx Ca levels as independent variables), BMI-T1 (p = 0.03) and pre-KTx albumin (p = 0.07) were the strongest independent determinants of LVMI (Table 4).

**Table 4:** Multivariate regression model: principal factors impacting on LVMI-T1.

Parameter	Std.Coeff	t-value	p-value
Dialysis vintage	0.11	1.11	0.26
BMI T1	0.22	2.18	**0.03**
Pre-KTx albumin	− 0.18	− 1.82	0.07
Pre-KTx Ca	0.16	1.68	0.09

Bold format: statistical significance (p < 0.05).

*BMI* body mass index.

#### LVH at T12

In 75 KTxp, EC evaluation was repeated at T12. In those patients, average LVMI was 51.5[39–63] g/m^2,7^ and it was not significantly different from LVMI-T1. LVH was found in 40 (52%) KTxp, the most prevalent LVH remodeling was C-LVH (27%). At univariate regression LVMI-T12 was directly correlated with age (p = 0.002), SBP-T12 (p = 0.0009), BMI-T12 (p = 0.01) and baseline LVMI (p < 0.0001). We did not observe any correlation of LVMI-T12 with renal function or Prot-U. Among mineral metabolism parameters we found a direct correlation of LVMI-T12 with PTH-T12 (p = 0.007) and alkaline phosphatase (ALP) at T12 (p = 0.0001) however we did not find any correlation of LVMI-T12 with 25OHD-T12.

A comparison between patients with LVH (LVH-T12pos) and those without LVH (LVH-T12neg) at T12. LVH-T12pos were older (p = 0.009), they had higher BMI-T12 (0.009), SBP-T12 (p = 0.004) and Prot-U-T12 (p = 0.03). LVH-T12pos and LVH-T12neg did not show any difference regarding: gender distribution, type of dialysis or KTx, N-VitD status and immunosuppressive therapies.

In multivariate regression model (LVMI-T12 as dependent variable; age, BMI-T12, Prot-U-T12 and PTH-T12 as independent variables), age (p = 0.02) and PTH-T12 (p = 0.01) were independent determinants of LVMI-T12 (Table 5).

**Table 5:** Multivariate regression model: principal factors impacting on LVMI-T12.

Parameter	Std.Coeff	t-value	p-value
Age at KTx	0.25	2.26	**0.02**
BMI T12	0.17	1.59	0.11
Prot-U T12	− 0.009	− 0.07	0.93
PTH T12	0.26	2.49	**0.01**

Bold format: statistical significance (p < 0.05).

*BMI* body mass index; *Prot-U* urinary protein excretion of the 24 h; *PTH* parathormone.

#### LVH modifications during the first year of KTx

At T12, 57 patients (76%) had a substantial stability of their LVH status in respect to T1 (LVH-stable), 11 pts (14%) developed LVH (LVH-prog), and 7 pts (10%) had a regression to a complete normalization of LVH (LVH-reg). Of note, among 57 LVH-stable KTxp, 36 were in LVH-T1pos group and maintained their status at T12 (LVH-T1pos/T12pos).

With the aim to evaluate the factors implicated in novel LVH development during the first year of KTx, the comparison between those two groups of patients was performed: 1) LVH-NO-prog (n = 28): LVH-reg (n = 7) + LVH-stable (n = 21; excluded LVH-T1pos/LVH-T12pos); 2) LVH-prog (n = 11). The decision to remove LVH-T1pos/T12pos from the analyses was derived by the potential statistical bias derived by their presence.

We reported in Table 6 a comparison between LVH-NO-prog and LVH-prog.

**Table 6:** General characteristics of the cohort studied according to LVHprog status.

Parameters	LVH-NO-prog (n = 28)	LVH-prog (n = 11)	p
Age (years)	39[30–52]	56[46–68]	**0.007**
Dialysis vintage (months)	35[14–60]	54[26–96]	0.29
Body Weight –T1 (Kg)	64[54–72]	71[66–78]	0.07
BMI –T1 (kg/m^2^)	22[19–25]	24[23–28]	0.06
Body Weight –T12 (Kg)	69[56–73]	73[67–76]	0.51
BMI –T12 (kg/m2)	23[20–26]	25[23–28]	0.22
SBP-T1 (mmHg)	130[120–140]	140[130–160]	0.11
DBP-T1 (mmHg)	80[71–87]	80[75–90]	0.84
SBP-T12 (mmHg)	125[112–137]	140[130–150]	**0.04**
DBP-T12 (mmHg)	80[70–85]	85[80–85]	0.37
s-creatinine-T1 (mg/dL)	1.34[1.0–1.6]	1.30[1.0–1.6]	0.99
s-creatinine-T12 (mg/dL)	1.36[1.2–1.5]	1.26[1.0–1.4]	0.09
Blood glucose-T1 (mg/dL)	79[68–86]	89[73–104]	0.14
Blood glucose-T12 (mg/dL)	89[73–104]	96[70–123]	0.12
Hb-T1 (g/dL)	11.3[9.7–12.0]	11.2[10.2–11.3]	0.97
Hb-T12 (g/dL)	12.9[12.1–13.6]	12.8[12.5–14.8]	0.08
s-albumin-T1 (g/dL)	4.30[3.9–4.6]	4.05[3.9–4.3]	0.11
s-albumin-T12 (g/dL)	4.60[4.5–4.7]	4.30[4.1–4.6]	**0.01**
PTH-T1 (ng/mL)	68[39–118]	52[35–64]	0.08
PTH-T12 (ng/mL)	60[44–96]	50[26–71]	0.11
Ca-T1 (mg/dl)	9.93[9.3–10.2]	9.67[8.9–9.8]	0.15
Ca-T12 (mg/dl)	9.93[9.5–10.2]	9.80[9.6–9.9]	0.37
P-T1 (mg/dL)	2.40[1.9–2.7]	3.10[2.5–3.5]	0.45
P-T12 (mg/dL)	3.10[2.4–3.6]	2.90[2.7–3.4]	0.86
ALP-T1 (U/dL)	84[63–103]	113[63–153]	0.20
ALP-T12 (U/dL)	74[54–95]	97[91–124]	**0.03**
25OHD-T1 (ng/dL)	13[6–15]	16[8–24]	0.19
25OHD-T12 (ng/dL)	20[14–27]	18[8–39]	0.96
m25OHD (ng/dL)	17[11–22]	20[8–25]	0.49
Prot-U-T1 (g/24 h)	0.0.16[0.12–0.26]	0.17[0.13–0.35]	0.39
Prot-U-T12 (g/24 h)	0.17[0.12–0.19]	0.18[0.12–0.30]	0.06
LVMI-T1 (g/m^2,7^)	40.5[32–45]	45.7[36–46]	0.35
LVMI-T12 (g/m^2,7^)	45.7[36–46]	61[52–75]	** < 0.0001**

Bold format: statistical significance (p < 0.05); t-test and Mann–Whitney test were used.

Continuous variables were expressed as median value and interquartile range [25%ile; 75%ile].

*BMI* body mass index; *SBP* systolic blood pressure; *DBP* diastolic blood pressure; *Hb* hemoglobin; *PTH* parathormone; *Ca* calcium; *P* phosphorus; *ALP* alkaline phosphatase; *Prot-U* daily urinary protein excretion; *LVMI* left ventricular mass index; “*s*” serum; *m25OHD* mean levels of native vitamin D.

LVHprog + were significantly older with worse SBP control. In addition, at T12, they were characterized by lower s-Albumin and higher alkaline phosphatase concentrations. LVHprog + had higher values of Prot-U at both T1 and T12. Renal function was not significantly different in the two groups. The two groups did not show any significant difference regarding 25OHD levels/status and other mineral metabolism parameters. No differences among LVHprog- and LVHprog + were found in gender, type of dialysis and KTx or in the general and immunosuppressive therapy.

In order to evaluate which factors might have influenced the new onset of LVH we made a logistic regression analysis (Table 7). In this analysis we chose LVH-prog as dependent variable, whereas we considered as independent variables: age, BMI-T12, Prot-U-T12 and s-Albumin-T12. Among them, only s-Albumin-T12 was significantly associated with LVH-prog (p = 0.04). Of note, the inclusion in the model of SBP-T12, resulted in a few reduction of s-albumin-T12 significance (p = 0.05).

**Table 7:** Multivariate logistic model: principal factors impacting on LVH-prog.

Parameter	Coef/SE	CI	p-value
Age at KTx	1.01	0.95;1.13	0.31
BMI T12	− 0.39	0.69;1.27	0.69
Prot-U T12	1.24	0.22;756	0.21
s-Albumin-T12	− 2.03	− 3.64;0.76	0.04

Bold format: statistical significance (p < 0.05).

*BMI* body mass index, *Prot-U* daily urinary protein excretion; “*s*” serum.

## Discussion

The aims of our research were to evaluate, in a cohort of KTxp followed up during the first year after KTx, the prevalence of native hypovitaminosis D and of LVH as well as to explore the eventual relationship between those factors.

In our cohort, at T1, the prevalence of i25OHD involved almost the totality of the KTxp. This is an important point considering that at T1, probably, the influence of KTx is still low both on clinical and biochemical factors, so it might be strongly influenced by previous status (dialysis or conservative management). Hypovitaminosis D is frequently found during CKD and its prevalence is proportional to the severity of renal impairment, reaching more than 85% in ESRD^9^.

The real prevalence of hypovitaminosis D in KTxp is a more debated subject. Recently published data have demonstrated that KTxp have higher levels of 25OHD than in ESRD patients (both hemodialysis and peritoneal dialysis patents)^10^. Nevertheless, as was recently reviewed by our group^11^, the evaluation of the prevalence of i25OHD in KTxp presents several difficulties and limitations. First of all, the different laboratory methods used in the published studies make the comparisons between Centers scarcely reliable. In our study we evaluated in our central laboratory all samples of 25OHD with the same method. In addition, the possible influence of seasonal variations or the modifications of lifestyle behaviors (outdoor or indoor activity) represents an aspect that is particularly relevant in KTxp and that might influence N-VitD status. Last but not least, it is important to underscore that, in the absence of clear and specific guidelines, some differences among transplant centers in N-VitD supplementation are certainly impacting. Unfortunately, up until now, only a few and methodologically contrasting studies explored the effect of i25OHD in KTxp. In our study, conducted during the first year of KTx, the prevalence of s25OHD increased by 2% to 15% but, despite N-VitD supplementation, median values of 25OHD at T12 were still significantly low. The prevalence of i25OH found in our cohort is consistent with the reports of some recent studies. Data from 419 Canadian KTx, in which a single KTx was performed over a 4-month period, showed s25OHD in 24.4% of the cohort studied^12^. European data, from France, report after 3 months of KTx only 8% of KTx with s25OHD ^13^. Despite the high prevalence of i25OH in transplanted population, actually the 25OHD supplementation is a therapy still under prescribed in clinical practice. This, in our opinion, is the reflection of some different factors. First of all, at the present time, no clear guidelines concerning the treatment of MM anomalies in KTx, especially after the first year of KTx^14^. Unfortunately, especially in the past, there was not sufficient sensibilization of the medical community to dose 25OH and to provide to its supplementation. In addition, the fact that native vitamin D supplementation is not a first line therapy for KTx (as immunosuppression) makes it not always considered as important by the patients and by their caregivers. In our opinion, the supplementation of native vitamin D should be done, when Ca levels allow, as a first line therapy in the treatment of MM anomalies in KTxps.

In agreement with what is reported in the literature, also our data confirm the strict and inverse relationship between 25OHD levels and PTH, present at both T1 and T12. The presence of i25OHD may then exacerbate the hyperparathyroidism status that is often present in the early phases of KTx^15^.

The second purpose of our study was to determine the prevalence of LVH in our cohort, evaluating also its modifications during the first year of KTx.

In our cohort, LVH was found in 42% of KTxp, and this result is in line with the data that were previously reported in the literature where it ranges between 36%- 77%. As it was for N-VitD, also for LVH the data obtained at T1 were mostly influenced by the pre-KTx period. This is clearly demonstrated by the fact that LVMI was correlated with: age at transplantation, dialysis vintage, SBP, BMI, Prot-U and pre-KTx albumin levels.

The correlation between dialysis vintage and LVMI is supported by a great deal of evidence. ESRD is characterised by several clinical and biochemical anomalies that may prompt the development of LVH. Anemia, high output arterial-venous dialysis accesses, pressure and volume overload (especially in anuric patients) might directly influence cardiac geometry and its consequent remodelling^16–18^. In addition, uremic status is characterised by demodulations in nitric oxide metabolism and by renin angiotensin system activity that may impact heart fibrotic processes^19,20^. Interestingly, the strong correlation between dialysis vintage and LVMI that was found at T1, was not confirmed at T12. This, possibly influenced by the consistently lower number of echocardiographic evaluations at T12, may indicate that: (1) the resolution of ESRD by KTx nullifies the effects of ESRD related factors; (2) some KTx related factors may influence the progression or the development of LVH.

At T12, the prevalence of LVH was 52%. At this time point we found some correlations that are consistent with some previous findings. We found that LVMI and LVH were correlated with BMI. Adipose tissue is nowadays considered an active organ, with high remodelling capacity and visceral obesity and an independent risk factor for LVH also in normotensive patients^21^. However, in our cohort KTxp were mostly normal-weight, thus probably, as evidenced in multivariate regressions, the effect of adiposity in influencing LVH-T12 and LVH progression might be of secondary relevance. A direct correlation between SBP at T12 and LVMI T12 and LVHprog + was reported. The not optimal SBP control might have represented one of the determinants of LVHprog +, considering also that no significant differences between anti-hypertensive therapy at T1 and T12 were present. Prospective and randomized trials on this topic might explore deeper this point in this kind of patients. The relationship between Prot-U and LVH was well explored in the general hypertensive population in 2004 by an Italian group. In their work an increase of 30% of the risk of cardiac damage was found for each 0.2-mg/mmol increase in Log albuminuria/creatininuria^22^. The relationship between Prot-U and LVH in KTxp has been investigated in only a few studies. In 2009 Paoletti et.al showed, a correlation between Prot-U and arterial hypertension with KTx organ damage^23^. More recently, in 2016, a significant correlation between Prot-U and LVMI has been proven in 1000 KTxp^24^. The most relevant hindrance in studying this relation in KTx patients lies in distinguishing the real clinical significance of Prot-U. Indeed, in KTxp proteinuria might depend on immunologic damage as well as on glomerular hypertension and subsequent sclerosis. In any case, in our cohort the second hypothesis was more likely to occur, considering that the levels of Prot-U are low and renal function is generally optimal. At T12, we found a de novo LVH in 11 patients (14%) and a regression of LVH in 7 patients. Among the factors that were evaluated, low s-Albumin level was the strongest factor determining LVH progression. Pre-KTx and T12 s-Albumin levels were inversely correlated to LVMI and LVH also in EC evaluations at T1 and T12. In dialysis patients several years ago Foley, et al. described a strong correlation between hypoalbuminemia and cardiac diseases^25^. More recently, Rigatto et al. reported that hypoalbuminemia plays a relevant role in the development of chronic heart failure in KTxp. Even though in ESRD the role of hypoalbuminemia in determining the development of LVH is well established, the persistence of this relationship in KTxp, especially in the absence of extracellular volume measurement, could not rule out the potential influence of fluid retention on heart work. It has still not been examined in depth and will probably deserve further attention in future studies. Some evidences reported in literature have recognized that that low albumin levels could be also a consequence of a subclinical chronic inflammatory status^26^. Unfortunately, in our work was not possible to study this point that might be the topic of future research. The third and last aim of our study was to explore the relationship and the eventual impact of N-VitD on LVH and LVH progression. Our data did not show any correlation between 25OHD levels / status and LVH. Generally, although animal studies have demonstrated that vitamin D supplementation may attenuate LVH, the results of human studies are frequently inconsistent. Furthermore, almost all those studies, none of which was conducted on KTxp, evaluated the relationship between i25OHD and cardiovascular diseases and they did not specifically address the effect of N-VitD on LVH^27^.

Recently, a randomized, placebo-controlled trial, performed in the general population (25,871 participants followed up for 5.3 years) has been published. Treatment consisted in 2000 IU of cholecalciferol per day and did not affect cardiovascular endpoints^28^. The results of the J-DAVID study were published in the beginning of 2019. In this randomized open-labels study performed in Japan, 976 haemodialyzed patients with PTH equal or less of 180 pg/mL were randomized to (1) treatment with oral alfacalcidol or (2) treatment without any VDRA. The overall cohort was followed up for a total of 48 months, and fatal/nonfatal cardiovascular events were considered as primary outcome. Also in this case, no reduction in cardiovascular events was demonstrated with oral alfacalcidol therapy^29^.

Our study has several limitations. First, it has a retrospective and observational design. In addition, it involves a relatively small number of KTxp, especially for what concerns the analyses of LVH progression. Levels of 25OHD at T1 and T12 were present only in 178 among the 670 transplanted in our centre between April 2004 and November 2017. The retrospective design of the study, and the variable importance given to this parameter over the years are probably the main determinants of this limitation.

Nevertheless, it is one of the few studies that focused its aim on the relation between N-VitD and LVH in KTxp.

In conclusion, in our retrospective observational study, performed in KTxp, we confirmed the high prevalence of N-VitD insufficiency, especially at the beginning of KTx and the scarce increase of 25OH levels despite supplementation during the first year of KTx.

LVH was found in about half of KTx p at T1 and T12 with some different relations between LVH and risk factors at the two time points. This observation might depend on the fact that some KTx specific factors may impact cardiac remodelling in KTxp.

Finally, we did not find any correlation between N-VitD levels/status and LVH or LVH variations.

We believe that overall our results represent a valuable basis to design future studies aimed at investigating the factors impacting LVH in KTxp.

## Methods

### Cohort Characteristics and study design

This study includes 178 KTx patients (M = 118; median age 49[39–59] years), among the 670 transplanted in our centre between April 2004 and November 2017, whose 25OHD levels at both T1 and T12 were available.

In addition to the parameters available and recorded at T1 and T12, for all the patients pre-KTx data concerning blood glucose, s-creatinine, Hb, serum albumin, PTH, Ca, P and Alkaline Phosphatase have been recorded. EC evaluation was available at T1 in all patients and at T12 in 75 individuals. Patients were followed up for the first year of KTx.

Patients that were included in our observational study were comparable in their characteristics to the overall cohort.

After KTx all patients were followed regularly at our outpatients clinic for the whole period of observation and they were treated in accordance with their clinical needs. The protocol was conducted according to the ethical principles of the Helsinki Convention and of the Declaration of Istanbul and it was presented and approved by the Ethical Committee of Fondazione IRCCS Ca’ Granda Ospedale Maggiore Policlinico of Milan.

### Biochemical evaluations

Data concerning biochemical analyses before KTx (Pre-KTx) were digitally recorded from the documents presented to each patient at the moment of pre-KTx evaluation.

After KTx, at T1 and T12, biological samples were collected in our Department after a fasting night. All biochemical analyses were performed in the same laboratory at our Institution.

Serum creatinine was determined by using Jaffe method whereas Prot-U was determined by immunoturbidimetric method.

PTH was measured with two different methods: until December 2013 it was determined with ECLIA (Elettro-Chemi-Luminescent Immuno-Assay) method by Roche and from January 2014 by DiaSorin LIAISON kit. Therefore we had to uniform all the determinations in accordance to the indications reported in the literature^30^. 25OHD levels were determined by enzyme-immunoassay (Kit EIA AC-57FI -immunodiagnostic system Boldon, UK), using a highly specific sheep 25OHD antibody and enzyme (horseradish peroxidase) labelled avidin. Mean 25OHD level (m25OHD) was calculated considering all the determinations performed between T1 and T12. Levels of 25OHD < 30 ng/dL were categorized as insufficient (i25OHD) and those ≥ 30 ng/mL as sufficient (s25OHD).

All other biochemical parameters were evaluated according to routine methodology used at the central laboratory of our Institution. All the biochemical results were digitally recorded.

### Subclinical cardiac damage evaluation

EC evaluation was performed at T1 in all patients and at T12 in 75 individuals. All exams were performed for routine clinical surveillance and not for a specific clinical indication. EC images were obtained by trained operators by means of standard M mode and two-dimensional images. In accordance with the recommendations of the American Society of Echocardiography we measured: end diastolic left ventricular internal diameter (LVIDd), diastolic posterior wall thickness (PWTd), and diastolic septal wall thickness (SWTd). Relative wall thickness (RWT) was estimated at end diastole and considered to be increased if > 0.45. According to the American Society of Echocardiography, left ventricular mass was calculated using simplified cubed equation. LVMI was indexed by height^2,7^ to normalize heart size to body size. LVH was defined as an LVMI > 51 g/m^2,7^ in both males and females.

Cardiac remodeling patterns were classified as follows: normal (absence of LVH and RWT < 0.45), concentric remodeling (C-Rem: absence of LVH and RWT > 0.45), eccentric hypertrophy (EC-LVH: LVH and RWT < 0.45), and concentric hypertrophy (C-LVH: LVH and RWT > 0.45).

LVH progression was defined by the development of LVH between T1 and T12 (absence of LVH at T1—presence of LVH at 12). LVH regression was defined by the resolution of LVH between T1 and T12 (presence of LVH at T1—absence of LVH at T12). Left ventricular stability was defined by the steadiness of LVH at T1 and T12 (presence of LVH at T1—presence of LVH at T12 or absence of LVH at T1—absence of LVH at T12).

### Statistical analysis

In statistical analyses, continuous variables were expressed as median value and interquartile range [25%ile; 75%ile] and were log transformed if they had a skewed distribution.

Differences among groups were determined by Student’s t test, Mann–Whitney, where indicated. Differences among percentages were determined by χ^2^ or Fisher test where indicated.

Linear regression and logistic/multiple regression were employed in order to perform uni-variated and multi-variated analysis respectively.

Statistical analysis was performed using software Statistica version 10 and SPSS version 20 and significance was set for p values < 0.05.

### Ethical approval

All procedures performed in studies involving human participants were in accordance with the ethical standards of the institutional and/or national research committee and with the 1964 Helsinki declaration and of the Declaration of Istanbul and its later amendments or comparable ethical standards.

### Informed consent

Informed consent was obtained from all individual participants included in the study.
